# Stem Cells as Potential Targets of Polyphenols in Multiple Sclerosis and Alzheimer's Disease

**DOI:** 10.1155/2018/1483791

**Published:** 2018-07-12

**Authors:** Ankit Tandon, Sangh Jyoti Singh, Rajnish Kumar Chaturvedi

**Affiliations:** ^1^Developmental Toxicology Laboratory, Systems Toxicology and Health Risk Assessment Group, CSIR-Indian Institute of Toxicology Research (CSIR-IITR), Vishvigyan Bhavan, 31, Mahatma Gandhi Marg, Lucknow, Uttar Pradesh 226001, India; ^2^Academy of Scientific and Innovative Research (AcSIR), CSIR-IITR Lucknow Campus, Lucknow, India

## Abstract

Alzheimer's disease (AD) and multiple sclerosis are major neurodegenerative diseases, which are characterized by the accumulation of abnormal pathogenic proteins due to oxidative stress, mitochondrial dysfunction, impaired autophagy, and pathogens, leading to neurodegeneration and behavioral deficits. Herein, we reviewed the utility of plant polyphenols in regulating proliferation and differentiation of stem cells for inducing brain self-repair in AD and multiple sclerosis. Firstly, we discussed the genetic, physiological, and environmental factors involved in the pathophysiology of both the disorders. Next, we reviewed various stem cell therapies available and how they have proved useful in animal models of AD and multiple sclerosis. Lastly, we discussed how polyphenols utilize the potential of stem cells, either complementing their therapeutic effects or stimulating endogenous and exogenous neurogenesis, against these diseases. We suggest that polyphenols could be a potential candidate for stem cell therapy against neurodegenerative disorders.

## 1. Introduction

Neurodegeneration refers to the progressive loss of neurons in the Central and Peripheral Nervous System (CNS and PNS). Genetic and environmental factors both play a vital role in the progression of neurodegenerative diseases including Parkinson's disease, Alzheimer's disease (AD), and multiple sclerosis (MS) [1]. Hallmarks of these diseases are impairment of Ubiquitin Proteasome Pathway and accumulation of pathogenic proteins in the discrete brain regions due to oxidative and nitrosative stress, mitochondrial dysfunction, and impaired autophagy [2]. Currently, no cure exists for these diseases, and available drugs provide only symptomatic relief. Thus further understanding of the pathophysiology of these diseases is essential for controlling the menace caused by these diseases. In this review, we will focus on two major neurodegenerative diseases, namely, AD and MS.

Neural stem cells (NSCs) are the cells of the brain that have the remarkable ability to develop into many different types of cells including neurons, astrocytes, and oligodendrocytes [3]. These cells are unspecialized cells that possess the property of self-renewal [3]. The main advantage of NSCs is that under certain physiological conditions these cells can be programmed to differentiate into neurons [4]. The NSCs hold a vast potential in the field of regenerative medicine in these debilitating diseases. Studies are being carried out frequently to tap this potential of NSCs against neurodegenerative disorders, with promising results [5].

Polyphenols comprise a set of naturally derived, synthetically synthesized, and semisynthetic organic chemicals characterized by the presence of multiple phenol structural units. They are mainly secondary metabolites of plants that are involved in defense and mostly present in fruits, vegetables, and cereals. They have immense benefits for health primarily due to their anti-oxidant properties. Many studies have highlighted their potential against a wide range of diseases [6]. In this review, we discuss the understanding of pathophysiology of AD and MS. Further we discuss how stem cells have proved their efficacy against these two diseases and finally how polyphenols can target stem cells for inducing brain self-repair or neurogenesis process (generation of new neurons) in AD and MS.

### 1.1. Alzheimer's Disease (AD)

AD is the most prevalent type of dementia characterized by the progressive decline in cognitive abilities of an individual [7]. Individuals aged 65 years or older are susceptible to this disease [8]. In the present scenario, AD accounts for nearly 50%-70% of the total dementia of which the higher age group accounts for the larger proportion [9]. According to 2012 WHO report on “Dementia: A Public Health Priority” approximately 35 million people are presently affected with dementia, and the frequency is expected to double by 2030 and triple by 2050 [10]. The association of the pathophysiology of AD is with the death of neurons originating in the hippocampus region of the brain, which gradually affects the entire brain [11]. The primary cause of AD is the abnormal accumulation of a short peptide amyloid beta (A*β*). A*β* originates by the proteolytic cleavage of transmembrane protein, amyloid precursor protein (APP). Genetic, environmental, and physiological factors are involved in the progression of the disease [12] (Tables 1–3).

### 1.2. Multiple Sclerosis (MS)

MS is primarily a neurodegenerative disease as it involves the progressive degeneration of the grey and white matter resulting in atrophy of the brain and spinal cord [13]. A 2015 study on the global prevalence of MS reported 2.3 million people affected by the disease [14]. MS is characterized by the presence of lesions or plaques predominantly in the white matter region of the brain and spinal cord [15]. It involves the loss of myelin sheath forming cells, termed oligodendrocytes, which helps in faster conduction of the nerve impulses (electrical signals) along neuronal axon [16]. While the cause of the disease remains elusive, it is primarily thought to be an autoimmune disorder [17]. MS is classified into relapsing-remitting MS and primary progressive MS, with the former being the more commonn.

Genetic, physiological, and environmental factors are involved in the progression of MS (Table 4). Presently there is no cure for MS, and treatments are available depending on the specific symptoms. As of 2012, monoclonal antibodies such as alemtuzumab, daclizumab, rituximab, ocrelizumab, and ofatumumab have shown positive results in clinical trials [18], of which FDA approved ocrelizumab for relapsing and primary MS in 2017. Estriol, a female sex hormone, has shown potential against relapsing-remitting MS in phase II trials [19].

## 2. Polyphenols Utilizing Stem Cells against Neurodegeneration

**Figure 1:**
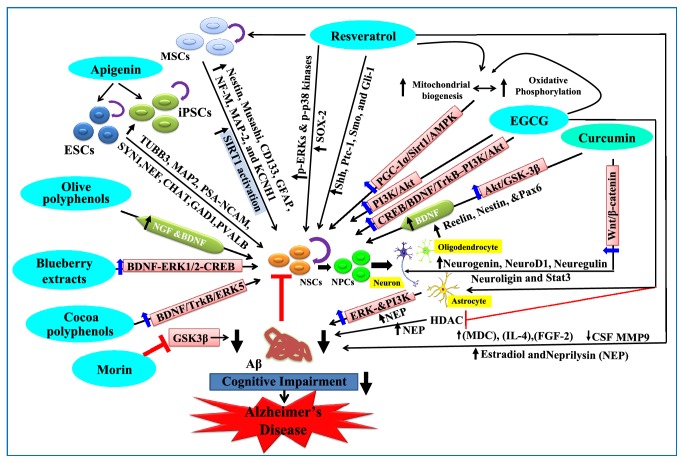
**The systematic representation for polyphenols targeting stem cells in AD.** Polyphenols rich diets, antioxidants, and vitamins play a quintessential role as a defensive tool for AD. Resveratrol, curcumin, olive polyphenols, blueberry extracts, cocoa polyphenols, and apigenin decrease A*β*-induced cellular changes by counteracting ROS through their anti-oxidant characteristics. Increased accumulation of A*β* in the brain causes synaptic dysfunction and mitochondrial dysfunction, leading to cognitive impairments. Resveratrol stimulates NPCs proliferation, thus accentuating high rates of neuronal survival by mediating SIRT1 activation. Resveratrol in conjunction with tea polyphenols EGCG modulates mitochondrial biogenesis which in turn restores the oxidative phosphorylation. EGCG exerts its neuroprotective action by increasing neuronal plasticity. Curcumin acts through two main pathways: firstly by inducing neuronal differentiation in NSCs through the activation of Wnt signaling and secondly by upregulating the genes required for the cell differentiation, respectively. Other polyphenols, e.g., apigenin, upregulate the levels of neurotrophic factors and expression of neuronal markers in iPSCs and ESCs and result in neuronal differentiation. Cocoa polyphenols, olive polyphenols, and blueberry extracts attenuate the toxic effects of A*β* deposition through increasing the levels of neurotrophic factors. The arrow line represents promoting effects, and the red line represents inhibitory effect.

**Figure 2:**
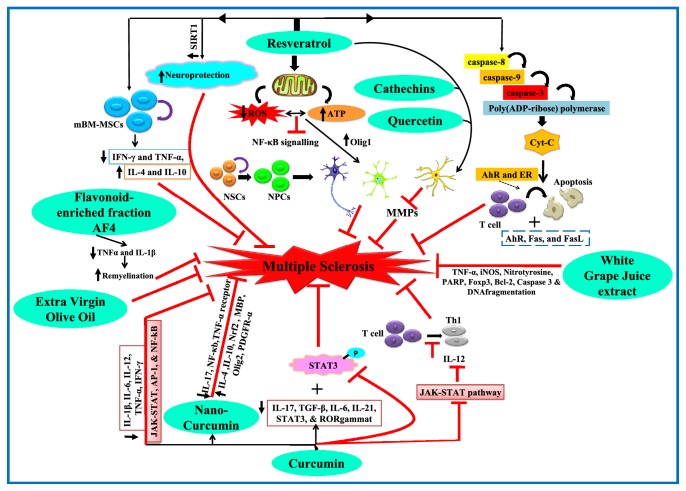
**The systematic representation for polyphenols targeting stem cells in MS.** In case of MS, the proinflammatory cytokines induce an obstructive effect on neural differentiation, whereas anti-inflammatory cytokines have an opposite action. The dietary antioxidants are associated with reduction of MS pathogenesis, by controlling the activation of inflammatory cytokines, reducing oxidative stress and apoptosis, and regulating migration and differentiation of stem cells. Polyphenols such as resveratrol, curcumin, flavanoid enriched fraction AF4, extra virgin olive oil, catechins, quercetin, and white grape juice extract showed neuroprotective potential in MS by targeting NSC proliferation and differentiation and reducing the elevated levels of inflammatory cytokines and oxidative stress. Resveratrol potentiates its protective action through AhR and ER that activates the T-cells causing apoptosis. Further, resveratrol exerts its neuroprotective role by reducing the oxidative stress and improving mitochondrial functions through inhibition of NF-*κ*B signaling which also results in remyelination. Quercetin and catechins along with resveratrol show an inhibitory effect on MMPs that inhibits cell migration and EGFR kinase activity.

Stem cells are undifferentiated, unspecialized cells characteristic of multicellular organisms that possess the potential of plasticity, that is, to develop many different cells of the body. Due to their ability to form new specialized cells, they are being studied thoroughly for their uniqueness in the area of translational medicine. Studies are being carried out worldwide to tap their potential in the field of neurodegeneration. Stem cell therapy against neurodegenerative diseases such as Alzheimer's, Parkinson, MS, amyotrophic lateral sclerosis, and Huntington disease is a promising area of research. Below we review the important role played by stem cells in AD and MS and how polyphenols can target the potential of stem cells in these disorders (Figures 1 and 2).

### 2.1. Targeting NSCs in AD

NSCs are the cells that give rise to neurons and other cells of the neuronal circuitry of the CNS by a process known as neurogenesis [3]. They are localized in the adult brain in two specific regions, namely, the hippocampal dentate gyrus (DG) and sub-ventricular zone of the ventricles (SVZ). The process of generation of new neurons from endogenous NSCs in the brain of AD patients and AD transgenic mice is found to be reduced. Therefore, NSCs could be targeted for enhancement of brain self-repair in AD. Several studies have implicated the role of NSCs in mitigation of AD pathology in mice. In hAPP transgenic mice expressing mutated human APP751, cerebrolysin treated NSCs were grafted, which led to increased levels of BDNF and furin and protection for neighbouring cells [20]. Genetically modified NSCs expressing A*β*-degrading enzyme neprilysin in 3xTg-AD and Thy1-APP transgenic AD mice models exhibited significant reduction in A*β* pathology and increase in synaptic density [21]. 192 IgG-saporin derived AD rats transplanted with embryonic rat NSCs induced by NGF-PEG-PLGA-nanoparticles facilitated differentiation of NSCs resulting in increased cholinergic neurons in basal forebrain regions, hippocampal synaptic formation, and generation of AChE-positive nerve fibers [22]. The APPsw-expressing (NSE/APPsw) transgenic AD mice transplanted with human NSCs exhibited improved spatial memory and increased neurotrophins (BDNF, NTF3, NTF4, NGF, VEGF, FGF2, and GDNF) levels, which resulted in decreased tau phosphorylation and A*β* production* via* Akt/GSK3*β* signaling [23]. NSC transplantation in the hippocampal region of APP/PS1 Tg AD mice demonstrated improved synaptophysin and growth-associated protein-43 (GAP-43) expression resulting in increased synapse density and integral mitochondrial structure [24]. Research has revealed that A*β* possesses proliferative effects on NSCs. In double-transgenic mouse model of AD co-expressing mutants of amyloid precursor protein (APPswe) and presenilin 1 (PSEN1dE9), an increase in expression of NSCs and neurogenesis marker genes was reported [25]. Inhibition of Akt hyperactivation and oleic acid-producing enzyme, stearoyl-CoA desaturase, rescued NSC proliferation impairments in AD brain [26]. Injection of NSCs in (APP/PS1) Tg AD mouse improved expression of N-methyl-d-aspartate (NMDA) 2B unit, synaptophysin, protein kinase C *ζ* subtypes (PKC*ζ*), tyrosine receptor kinase B (TrkB), and BDNF proteins related to cognitive function [27]. Improvement in number of doublecortin (DCX) positive neurons in the dentate gyrus region in human NSC-transplanted Tg2576 AD mice was observed [28]. Stem cell factor (SCF) and its receptor c-kit, involved in the migration of NSCs to sites of brain injury, are expressed on NSCs [29]. Epigenetic factors also play a crucial role in regulating stem cell proliferation and differentiation [30]. HDAC inhibitors regulate histone acetylation in APP/PS1 transgenic mice increasing the generation of new neurons [31]. Similarly morphogens such as mitogen-activated kinase 1/extracellular signal-regulated kinase 2 and orphan nuclear receptor TLX support adult NSC proliferation through epigenetic mechanisms [32, 33]. These studies suggested that NSCs can be stimulated for proliferation and neuronal differentiation to mitigate cognitive dysfunctions in AD.

### 2.2. Targeting Embryonic Stem Cells (ESCs) in AD

ESCs are pluripotent cells derived from the inner cell mass of a blastocyst that possess the capability to differentiate into any cell type and exhibit remarkable long-term proliferative potential. Generation of neurons from ESCs is a promising approach to cell-based replacement therapies [34]. Several factors possessing the ability of neuronal differentiation from ESCs have been identified. The sonic hedgehog (Shh), retinoic acid, and leukemia inhibitory factor (LIF) increase cholinergic differentiation of neuronal cells derived from ESCs [35]. Retinoic acid has been shown to direct the differentiation of human ESCs into basal forebrain cholinergic neurons (BFCNs) [36]. The NKX2-1 is essential for maturation and maintenance of BFCNs derived from NKX2-1^+^ ventral telencephalic progenitors [37]. Differential processing of A*β*PP regulates hESC proliferation and differentiation into NPCs [38]. NGF is essential for maintaining cholinergic phenotype, cholinergic synaptic integrity, regulation of dendritic growth, axonal guidance, long-term potentiation (LTP), synaptic plasticity, neurotransmitter release, and induction of expression of cholinergic markers* via* the PI3-K/Akt pathway in ESCs derived BFCNs [39]. A switch from ESCs to NSCs requires expression of Oct4^+^, nanog^+^, Sox2^+^, Sox1^−^, Sox3^−^ to Sox1^+^, 2^+^, 3^+^, blbp^+^, hes5^+^, Oct4^−^, and nanog− markers, respectively [40]. Inhibition of BMP by chordin, noggin, and follistatin in association with signaling from bFGF helps to generate neural precursors, while bHLH family proteins such as Mash1, Math1, NeuroD, and neurogenin 1–3 help to induce differentiation of neurons and other cell types [41]. The hormones also play a crucial role in directing the conversion of ESCs into neuronal phenotype. Human chorionic gonadotropin and progesterone hormones provoke the proliferation and differentiation of hESCs* via* P4 receptor A, resulting in induction of neurulation [42]. It has been reported that progesterone mediated modulation of A*β*PP processing drives the conversion of hESCs into NPCs [38].

### 2.3. Targeting Mesenchymal Stem Cells (MSCs) in AD

MSCs are multipotent stromal cells present in the adult bone marrow that can differentiate into a variety of cell types under appropriate conditions [43]. In A*β*-treated SH-SY5Y cells, MSCs increased autophagy induction through an increase in beclin (BECN1) and LC3-II expression, enhancing fusion of autophagosomes and lysosomes and promoting A*β* clearance and survival of hippocampal neurons [44]. Transplantation of human umbilical cord MSC-derived neuron-like cells caused reduction in A*β* deposition, reduced microglial activation and neuroinflammation, and improved cognitive functions in AD transgenic mice model [45]. MSC transplantation resulted in decreased levels of potentially toxic A*β∗*56 and preserved levels of glutamine synthetase in a 3xTg-AD mouse model [46]. Similarly, transplantation of human umbilical cord blood (hUCB) MSCs into the hippocampus of mouse AD model reduced A*β* plaques, increased endogenous adult hippocampal neurogenesis and synaptic activity, and enhanced cognitive function [47]. Adipose-derived MSCs enhanced endogenous neurogenesis in APP/PS1 AD mice [48]. Transplantation of MSCs into A*β*_1–42_-infused mice activated glial cells, inhibited iNOS and COX-2 expression, downregulated the release of inflammatory cytokines, prevented neuronal cell death, promoted neuronal cell differentiation, and improved cognitive impairment [49]. MSC-VEGF treatment in 2xTg-AD APPswe/PS1dE9 mouse model favored neovascularisation and diminished senile plaques in the hippocampus, provided behavioral benefits, and reduced cognitive deficits [50]. In a study on hUCB-MSCs with BV2 microglia under A*β*42 exposure, it was observed that ICAM-1 was released from hUCB-MSCs by coculturing with BV2 cells, and it induced neprilysin (NEP) expression in microglia through sICAM-1/LFA-1 signaling pathway resulting in a decrease in A*β* plaques [51]. In a transgenic mouse model of AD, MSC-educated T-regulatory cells significantly improved impaired cognition and reduced A*β* plaque deposition followed by decreased levels of activated microglia and systemic inflammatory factors [52]. In PC12 cells treated by A*β*_25-35_, BMSCs inhibited apoptosis via TAG1/APP/AICD signal pathway [53]. Thus these studies show the protective action of MSCs against AD pathogenesis.

### 2.4. Targeting Induced Pluripotent Stem Cells (iPSCs) in AD

The iPSCs are cells generated directly from adult somatic cells by introducing products of specific sets of pluripotency-associated genes into a given cell type. Thus they hold great promise in the field of regenerative medicine [54]. Several studies have shown their potential in many neurodegenerative disorders [54]. In iPSCs, PI3K-Akt represents a crucial survival signaling pathway [55]. The iPSC-derived NSCs transplanted in AD model show reduced tau phosphorylation, BACE1 expression, decreased expression of inflammatory mediators, and reduced A*β* production* via* Akt/GSK3*β* signaling [56]. In human iPSCs, the Mfn2 expression is essential for the maintenance of morphology and functioning of mitochondrial network and mitochondrial metabolism, the altered expression of which promotes AD pathology [57]. In human iPSCs, it has been shown that intracellular signaling molecules BDNF and cAMP can induce SORL1 expression demonstrating a significant reduction in A*β* [58]. Studies have shown that in protein-induced iPSCs, apotransferrin significantly promoted the maturation of oligodendrocyte differentiation and decreased plaque depositions in the 5XFAD transgenic AD mouse model [59]. Neurogenin 2 (NGN2) is a proneural transcription factor which is essential for the induction of neurons from iPSCs thereby ameliorating AD pathogenesis [60]. Thus, regenerative and modeling potential of iPSC present it as an indispensable tool in AD therapeutic research.

### 2.5. Polyphenols Complementing Stem Cell Therapy and Reducing Neurodegeneration in AD

Studies have highlighted the protective role of polyphenols against a wide range of disorders. Here we present a review of studies highlighting the protective effects of different polyphenols against AD. In a study, it has been reported that cocoa polyphenols protect cell viability and cell morphology against A*β* injury and promote neuronal survival* via* neuronal survival pathway BDNF/TrkB/ERK5 [61]. Olive polyphenols increased the levels of NGF and BDNF in limbic system and olfactory bulbs promoting proliferation and migration of endogenous progenitor cells in mice [62]. Green tea polyphenol epigallocatechin gallate (EGCG) effectively inhibited sevoflurane-induced neurodegeneration and improved learning and memory ability in mice* via* activation of CREB/BDNF/TrkB–PI3K/Akt signaling [63]. In A*β* infused rats it has been shown that curcumin caused neuroprotection* via* activation of Akt/GSK-3*β* signaling pathway and increased expression of BDNF [64]. In APP/PS1 transgenic AD mice fed with blueberry extracts, it was shown that BDNF-ERK1/2-CREB pathway was involved in alleviation of neuronal loss, enhancement of hippocampal neuronal plasticity, and improvement of cognition and memory [65]. In AD mice model, resveratrol significantly increased estradiol and neprilysin levels, which resulted in decrease in A*β* deposition and reversal in decline of memory [66]. In SAMP8 AD mice, EGCG rescued cognitive deterioration and induced reduction in A*β* accumulation* via* elevated neprilysin expression [67]. In rat AD model, EGCG facilitated A*β* degradation by increasing astrocyte secretion of neprilysin* via* activation of ERK- and PI3K-mediated pathways [68]. Green tea extracts lead to an increase in neprilysin activity and degradation of plaque-forming peptides in SK-N-SH neuroblastoma cell line [69]. Isorhamnetin (flavonol aglycone isolated from the leaves of* Ginkgo biloba L*.) potentiated NGF induced neurite outgrowth and increased expression of neurofilament in PC12 cells [70]. In one clinical study on the human subjects with mild to moderate AD, resveratrol significantly reduced cerebrospinal fluid (CSF) MMP9 levels and increased macrophage-derived chemokine (MDC), IL-4, and FGF-2 levels (Trial Registration: ClinicalTrials.gov NCT01504854) [71]. EGCG enhanced neuroregeneration* via* significant increase in the gene expression of growth factors FGF2 and VEGF in male rats with spinal cord injury [72]. Quercetin promotes neurite growth by significantly increasing the expression of cellular cAMP and GAP-43 in neuroblastoma N1E-115 cells [73]. EGCG and resveratrol restore oxidative phosphorylation and mitochondrial biogenesis and improve proliferation of NPCs* via* the activation of PGC-1*α*/Sirt1/AMPK axis in Ts65Dn mouse model of Down syndrome [74]. Resveratrol rescued the decrease in the number of Sox2 positive hippocampal neuron progenitors in C57/BL6 mice [75]. Treatment of NSCs with apigenin resulted in increased number of NPCs and induced expression of neuronal markers and increased neurogenesis [76]. Resveratrol reversed lipopolysaccharide-induced decreased Sox2 expression in hippocampal NSCs [77]. In the hippocampus of aged rats, resveratrol stimulated NPCs proliferation mediated by increased phosphorylation of ERKs and p38 kinases. Further, resveratrol increased the number of newly generated cells in the hippocampus with up-regulation of p-CREB and SIRT1 proteins responsible for neuronal survival and lifespan extension, respectively [78]. Curcumin has been shown to promote neurogenesis by increasing the expression of reelin, nestin, and Pax6 (genes involved in cell proliferation) and neurogenin, neuroD1, neuregulin, neuroligin, and Stat3 (genes involved in cell differentiation)* via* activation of the Wnt/*β*-catenin pathway in AD rat model [79, 80]. Resveratrol increased the expression of neuron-specific genes nestin, Musashi, CD133, GFAP, NF-M, MAP-2, and KCNH1 and exhibited neuroprotection* via* SIRT1 activation in hBM-MSCs [81]. EGCG, an HDAC inhibitor reduces A*β* accumulation and elevates NEP expression, thereby rescuing cognitive deterioration in SAMP8 mice [67]. In NSE/hAPP-C105 Tg mice, GTC treatment was shown to activate Wnt signaling and rescue neurodegeneration in the brain [82]. In the developing rat brain, BPA-mediated impaired neurogenesis and cognitive dysfunctions were rescued by curcumin* via* activation of the Wnt/*β*-catenin signaling pathway [83]. In one study, it was reported that flavonoid morin attenuated A*β*-induced tau phosphorylation* via* inhibiting GSK3*β* activity, both* in vitro* and* in vivo* [84]. In mice it was shown that EGCG caused up-regulation of Shh receptor and Shh transcriptional target Gli1, which resulted in improved hippocampal neurogenesis and spatial cognition [85]. Similarly in rats, after ischemic cerebral stroke, resveratrol pre-treatment significantly increased expression of Shh, Ptc-1, Smo, and Gli-1 mRNAs, thereby improving neurological function, enhancing vitality, and reducing apoptosis of neurons [86]. Thus polyphenols have a wide scope in regenerative medicine by targeting factors responsible for stem cell proliferation and differentiation, against AD.

### 2.6. Targeting NSCs in MS

Several studies have highlighted the potential role of NSCs in the treatment of MS pathology. The pre-treatment with vitamin D depicted positive effect in triggering NSCs, thereby preventing the development and progression of disease and also hindering apoptosis in a mice model of MS [87]. The inhibition of Gli1 led to the mobilization of endogenous NSCs for the repair of demyelinated lesions in a relapsing/remitting model of RR-EAE [88]. In C57/BL6 mice with EAE, administration of glycosyltransferase-programmed stereo-substitution NSCs (GPS-NSCs) resulted in markedly decreased inflammation and improved oligodendroglial and axonal integrity [89]. Activation of either the parenchymal oligodendrocyte progenitor cells (OPCs) or the sub-ventricular zone-derived NSCs to ameliorate cognitive disability is a promising perspective for future therapy of MS [90]. Natural killer cells contribute to the rejection of allogeneic NPCs through an NKG2D/RAE-1 signaling pathway in a viral-induced demyelination model [91]. In C57BL/6 and Thy1-YFP mice infected intracranially with plaque-forming units of JHMV strain J2.2v-1, NPCs engrafted into spinal cords exhibited diminished migration velocities and increased proliferation [92]. NPCs particularly accumulated within areas of axonal damage, initiating direct contact with axons and consequently expressing the myelin PLP gene, thereby initiating remyelination [92]. Treatment with minocycline, an inhibitor of microglia activation, was shown to increase stem cell proliferation in both naïve and EAE animals, thereby improving the proliferation and differentiation of Sox2 stem cells and NG2 oligodendrocyte precursor cells originating in the SVZ [93]. Transplantation of PC12-derived neural-like cells into the brain lateral ventricles of EAE mice showed attenuation of the inflammatory process, thereby resulting in the reduction of both axonal damage and demyelination [94].

In primary progressive and secondary progressive MS patients, the CNS environment stimulated the endogenous pool of NPCs to differentiate into neurons and oligodendrocytes [95]. NSCs treated with 1,25(OH)2D3 showed elevated levels of neurotrophic factors vital for neural cell survival and differentiation, thereby exhibiting a direct effect of 1,25(OH)2D3 on NSC proliferation, survival, and neuron/oligodendrocyte differentiation, remyelination, and neuroprotection in MS [96]. Overexpression of Zfp488, the oligodendrocyte-specific zinc finger transcription repressor, has been shown to increase the differentiation of SVZ NPCs into oligodendrocytes within the demyelinated corpus callosum, thereby promoting functional recovery [97]. Electromagnetic fields have been shown to significantly reduce the extent of demyelinated area and increase the levels of MBP thereby potentiating proliferation and migration of NSCs and enhancing the repair of myelin in demyelination [98]. Overexpression of NFIX in NSCs has been shown to inhibit oligodendrogenesis and loss of NFIX significantly increased the number of oligodendrocytes derived from SVZ-NSCs [99]. Similarly, transcription factor Prox1 drives NSCs oligodendrogenesis in the SVZ [100]. Deletion of neurofibromin-1 was found to direct ectopic oligodendrogenesis from SGZ-NSCs [101]. CNTF influences the migration of NSC progeny toward the demyelinated corpus callosum* via* activation of the JAK/STAT3 pathway [102]. The transcription factor Ascl1/Mash1 plays a significant role in neurogenesis, and its deletion leads to loss of neuronal progenitors [103]. In both the corpus callosum and spinal cord, Emx1-expressing dorsal SVZ-NSCs were found to undergo increased proliferation, recruitment, and differentiation into oligodendrocytes in response to demyelination [104].

The CXCL8 expression in the CNS has been shown to drive recruitment of NSCs and OPCs to the sites of inflammation [105]. In EAE rodents it was observed that selectively blocking CXCR4, CCR2, or c-Met partially inhibited NPCs migration while blocking all three receptors had an additive effect and resulted in significant inhibition of NPCs migration in EAE brains [106]. In a diphtheria-toxin inducible genetic model for demyelination, Ascl1-mediated conversion of hippocampal NSCs into mature oligodendrocytes enhances remyelination [107]. Bioliberation of gold ions from metallic gold implants in an EAE rodent model of MS showed reduced apoptotic cell death, upregulation of neuroprotective proteins Metallothionein-1 and -2 in the corpus callosum, significant upregulation of NSCs migrating from the SVZ, and significant slowing of disease progression [108]. FTY720 (fingolimod), an FDA-approved drug for the treatment of MS, has been shown to exhibit survival and differentiation of NPCs, as well as remyelination and repair following brain injury [109]. It was found that LINGO-1 is a negative regulator of OPCs differentiation whereas RXRs are positive regulators of OPCs differentiation [110]. In one study on Focal experimental Auto-immune encephalomyelitis (fEAE), valproic acid administration resulted in increased recruitment of NSCs and oligodendrocyte precursors within the lesion leading to an increase in the number of remyelinated axons [111]. In another study it has been reported that IL-17 blocks proliferation of NSCs, resulting in significantly reduced numbers of OPCs, thereby blocking remyelination and neural repair in the CNS [112].

Genetically engineered NSCs programmed to produce therapeutic cocktail comprising IL-10, NT-3, and LINGO-1-Fc mediated recovery through inducing M2 macrophages/microglia, reducing astrogliosis, and promoting axonal integrity and endogenous oligodendrocyte/neuron differentiation, thereby demonstrating a new and potentially efficient therapy against the chronic stage of MS [296]. NO-induced neuronal to glial fate conversion is shown to rely on transcription factor NRSF/REST, a key factor in NPCs specific response to innate immunity, and suggests a novel mechanism by which signaling from inflamed tissue induces the formation of glial cells [297]. The dbcAMP has been shown to suppress EAE progression, decrease the extent of demyelinated plaques, and increase the recruitment of NSCs into the olfactory bulb and brain parenchyma of EAE mice [298]. The PDGFRA/NG2-expressing glia, a dispersed population of stem/progenitor cells in the adult CNS, generates oligodendrocytes and Schwann cells in chemically induced demyelination in mice [299]. In EAE mice, adult NG2^+^ cells represent a valuable cell population for initiation of neural repair in demyelinating diseases such as MS [300]. The CXCL12:CXCR4 pathway has been shown to be involved in regulating engrafted NSCs to sites of tissue damage within the CNS of JHMV mice model of MS [301]. In TMEV murine MS model, loss of galectin-3 (Gal-3) inhibited an increase in chemokine levels, reduced immune cell migration into SVZ, restored SVZ proliferation, and increased the number of progenitors in the corpus callosum, thereby modulating the SVZ neurogenic niche's response to MS [302]. A study reported a dose-dependent response of hNPCs to IFN *β*-1b treatment* via* sustained proliferation and differentiation in MS [303]. In a study, intraventricularly injected Olig2-NSCs significantly reduced the clinical signs of acute and relapsing disease, thereby ameliorating CREAE in mice MS model [304]. In chronic/non-remitting EAE MS model, a reduction in NSCs/NPCs proliferation in the SVZ and hippocampal subgranular zone of the dentate gyrus was reported [305]. Olig1 function has been reported to be essential for the remyelination potential of NPCs after transplant in JHMV MS model [306]. Olig2-transfected NSCs induced development of fully mature oligodendrocytes expressing the transcription factor NKX2-2 and all major myelin-specific proteins, thereby developing into actively remyelinating oligodendrocytes [307]. NG2-targeted LIF-nanoparticles bound to OPCs activated pSTAT-3 signaling and induced OPC differentiation into oligodendrocytes [308]. In a model of CNS demyelination, NG2-targeted LIF-nanoparticles increased myelin repair, the number of myelinated axons, and thickness of myelin per axon [308]. SIRT1 inactivation, mediated at least in part by Akt and p38 MAPK-signaling molecules downstream of PDGFR*α*, has been shown to increase oligodendrocyte expansion, thereby ameliorating remyelination [309]. FGF8 is a novel factor shown to induce OPCs activation, migration, and proliferation [310].

The Shh Gli1 pathway is an important signaling pathway for NSCs in EAE and MS [311]. Mild hypoxia has been shown to favor NSCs proliferation and neuronal and oligodendroglial differentiation in neurodegenerative disorders [312]. Donepezil has been shown to promote oligodendrocyte differentiation and myelin-related gene expression* via* nAChRs in NSCs-derived OPCs [313]. Grafted OPCs migrate toward areas of inflammation in the adult rat spinal cord, where they survive and differentiate into myelinating oligodendrocytes [314]. CCL19 has been shown to abolish OPCs differentiation observed in patients with high remyelination pattern [315]. In the presence of Ephrin-B3, a transmembrane signaling protein, OPCs fail to differentiate, thereby inhibiting remyelination. Thus Ephrin-B3 in MS lesions inhibits OPC differentiation, while antibody-mediated masking of Ephrin-B3 epitopes promotes it [316]. FGF2 enhances hippocampal myelination and potentiates the recruitment of OPCs and NSCs to the lesion area [317]. Indomethacin, a non-steroidal anti-inflammatory drug (NSAID) modulates the Wnt/*β*-catenin pathway and thereby promotes differentiation of human and murine oligodendrocytes and remyelination in cuprizone-induced demyelination MS model [318]. IL1*β* and CCL2 enhance the mobilization of OPCs, thus contributing to remyelination [319]. Expression of PH20, a hyaluronidase, inhibits OPC maturation and remyelination, the pharmacological inhibition of which promotes remyelination in MS [320]. Galanin, a bioactive neuropeptide widely distributed throughout the nervous system, is a regulator of myelination and one of the myelination promoters [321]. Klotho, an anti-aging protein, increases OPC maturation and represents a useful therapeutic target in efforts to protect brain myelin against age-dependent changes and promote repair in MS [322]. Anti-EGF Ab treatment ameliorates EAE* via* induction of neurogenesis and oligodendrogenesis [323]. The increased Myt1 expression in both the periplaque white matter adjacent to lesions and within early remyelinating lesions was observed in MS lesions, suggesting a potential role for Myt1 in the regeneration of oligodendrocyte in response to demyelination [324]. Following activation by TNF, TNFR2 initiates pathways that drive oligodendrocytes generation and remyelination; thereby TNFR2 serves as a novel therapeutic target in MS [325]. Exogenous addition of ganglioside GD1alpha overcomes the inhibiting effect of aggregated fibronectin on OPC maturation, both* in vitro* and* in vivo*, by activating a PKA-dependent signaling pathway, thereby acting as a potential novel molecular tool against MS [326]. Fluvoxamine, an antidepressant drug, stimulates proliferation and differentiation of NSCs particularly toward oligodendrocytes in animal model of MS [327]. Thus these studies suggest a prospective role of NSCs therapy against MS disease.

### 2.7. Targeting MSCs in MS

Clinical trials using MSCs therapy in MS patients have shown tolerability and safety [328]. MSC transplantation is an approach to regulate the immune system in MS [329]. In relapsing-remitting MS patients bone marrow MSCs proved to be safe and reduced inflammatory parameters (ClinicalTrials.gov Reg. No. NCT01228266) [330]. Similarly, autologous MSCs given to patients with secondary progressive MS showed structural, functional, and physiological improvement suggesting neuroprotection (ClinicalTrials.gov, Reg. No. NCT00395200) [331]. The human umbilical cord MSCs treatment demonstrated high potential in MS patients [332]. Mice treated with human bone marrow-derived MSCs showed inhibition of EAE onset with decreased demyelination in the lumbar spinal cord and enhancement of immunomodulatory effects, which suppressed proinflammatory cytokines IFN-*γ* and TNF-*α* and increased anti-inflammatory cytokines IL-4 and IL-10 levels [333]. SJL-AdMSCs have been shown to ameliorate EAE course and modulation in disease progression [334]. Active MSCs condition medium containing HGF promoted recovery in EAE and markedly accelerated remyelination [335]. Intraperitoneal administration of MSCs expressing vasoactive intestinal peptide (VIP) stopped progression and reduced symptoms in EAE when administered at the peak of disease [336]. Interestingly, MSCs did not exert regenerative functions in EAE where peripheral immune cells and T lymphocytes were not involved, implying that the peripheral immune system is required for MSC to exercise their effects [337].

Combination of MSCs-IFN-*β* and minocycline on EAE mice considerably alleviated the clinical severity by sustaining the integrity of blood-spinal cord barrier through inhibition of microvascular disruption, neuroinflammation, and augmentation of immunomodulatory effects [338]. Human embryonic stem cell-derived mesenchymal stem cells (HES-MSCs), as compared to bone marrow MSCs, have been shown to significantly reduce clinical symptoms and prevent neuronal demyelination in a mouse EAE model of MS [339]. Transplantation of MSCs has been shown to stop the development of EAE by affecting CD4^+^, CD25^+^, Foxp3^+^, T-cell, Foxp3, TGF-*β*1, and IL-10 [340]. Therapeutic potential of bone marrow MSCs for MS is dependent on their immunosuppressive and immunomodulatory nature and their ability to enhance endogenous repair and remyelination [341]. In EAE mice, intrathecal injection of MSCs-derived neural progenitors improved neurological function with reduced immune cell infiltration, reduced area of demyelination, and increased number of endogenous NPCs [342]. EAE mice transplanted intracerebrally with human placental MSCs caused a decrease in disease severity by reduction of anti-inflammatory proteins [343]. In EAE rats treated with adipose-derived-MSCs, the human HLA-G gene was significantly expressed in the brain, thereby demonstrating striking therapeutic effects and unique immunomodulatory capacities [344]. Co-administration of 17*β*-estradiol (E2) and adipose-derived MSCs in a mouse cuprizone model of MS showed increased expression of Iba-1, Olig2, and O4 and enhanced efficacy in remyelination [345].

MSCs induced to differentiate into neurotrophic factor-generating cells exhibited the ability to suppress immune cells and protect neuronal cells from oxidative abuse, thereby delaying the symptoms of EAE in mice [346]. MSCs differentially modulate CD8^+^ T-cell development leading to worsening of EAE disease [347]. Aspirin treatment upregulates telomerase activity and stimulates TERT to improve the immunomodulatory capacity of bone marrow MSCs [348]. Inhibition of autophagy by knockdown of beclin-1 was found to significantly alleviate the therapeutic effects of MSCs in EAE model [349]. The ability of MSCs to cross oligolineage boundaries between mesodermal and ectodermal lineages leads to their differentiation into Schwann cells [350]. Transplantation of hWJ-MSCs-derived OPCs significantly reduced the clinical signs of EAE and enhanced remyelination [351]. MSCs can prime NPCs to the oligodendroglial fate leading to the formation of CNPase and MBP expressing oligodendrocytes and reducing the anti-oligodendrogenic determinant Id2 in proliferating NPCs [352]. The bone marrow hMSCs reduced interferon gamma-producing inflammatory cells, but increased IL-4 producing anti-inflammatory cells, thereby increasing oligodendrocyte lineage in EAE mouse model of MS [353]. In MS patients, after MSCs therapy, the expression of Foxp3 was reported to be significantly higher than the levels before treatment associated with clinical stability [354]. Co-transplantation with MSCs increased OPCs engraftment, migration, and maturation in myelinating oligodendrocytes, which produced widespread myelination in the host corpus callosum while reducing microglia activation and astrocytosis in the brain of transplanted animals [355]. Transplantation of Adi-IL-10-MSCs delayed the development of EAE with the reduction in peripheral T-cell proliferative responses, a decrease in proinflammatory cytokine secretion, and inhibition of Th17-mediated neuroinflammation [356]. Genetically modified AD-MSCs expressing murine interferon beta (MSCs-VP/IFN-*β*) showed significant induction of Tregs and IL-10 and reduction of IL-17 in EAE MS model [357]. Similarly, administration of Ad-IL4-MSCs in mice with EAE attenuated clinical symptoms leading to a decrease in peripheral MOG-specific T-cell responses and a shift from a pro- to anti-inflammatory cytokine response [358].

Combination of human bone marrow MSCs and minocycline in EAE promoted immunomodulatory effects, which suppressed proinflammatory cytokines IFN-*γ* and TNF-*α* and increased anti-inflammatory cytokines IL-4 and IL-10, thereby providing novel therapeutic potential against MS [359]. NG2^+^ cells stimulated endogenous repair by differentiating into oligodendrocytes, thus promoting neural repair in MS [300]. The umbilical cord blood MSCs-derived NPC reduced CNS leukocyte infiltration in EAE MS model [360]. RANTES and IP-10 promote hMSCs proliferation in MS lesions [361]. Treatment of EAE mice with MSCs engineered with PSGL-1/SLeX/IL-10 exhibited a superior therapeutic potential [362]. SOD3 secretion by MSCs holds potential in the treatment of MS [363]. MSCs showed antioxidant and neuroprotective activity in EAE MS model [364]. Treatment of EAE mice with the secreted ectodomain of sialic acid-binding Ig-like lectin-9 and SHED-CM is a novel therapy for MS [365]. In EAE, maintenance of GSK3*β* in its inactive status plays a role in preserving the normal physiology of the spinal cord and amelioration of EAE following MSCs therapy [366]. Combined treatment of Methylprednisolone and MSCs-IFN*β* had enhanced therapeutic effects on EAE mice [367]. Nicotine has been shown to augment the beneficial effects of MSCs based therapy in the rat model of MS [368]. Addition of rapamycin to bone marrow MSCs ameliorated neurological deficits and provided neuroprotective effects in EAE [369]. Pro-oligodendroglial factors derived from human fetal MSCs instruct human iPSC-derived NSCs to differentiate into O4 positive oligodendrocytes [370]. Thus the above studies highlight the important therapeutic role of MSCs in MS.

### 2.8. Targeting HSCs in MS

HSCs have proved to be a potential therapeutic tool against MS pathology. Autologous stem cell transplantation has proved to be beneficial in progressive forms of MS [371]. Autologous HSCs transplantation has been shown to completely halt disease activity in the majority of patients with aggressive MS [372]. In 3 patients with clinical MS, reconstitution of hematopoiesis and functional improvements were achieved with CD34-enriched stem cells even after withdrawal of all immunosuppressive medications [373]. In one study it was reported that HSCs expand regulatory cells and deplete IL-17 T-cells in MS [374]. Granulocyte colony-stimulating factor and cyclophosphamide resulted in an efficient HSC mobilization in MS patients [375]. In patients with relapsing-remitting MS, HSCs transplantation resulted in improvement in neurological disability [376]. In a patient suffering from large granular lymphocyte leukemia and concomitant primary progressive MS, allogeneic HSCs resulted in marked improvement in neurological conditions [377]. Autologous hematopoietic stem cell transplantation (AHSCT) restores immune tolerance in MS patients through increased numbers of regulatory T-cell subsets and PD-1 inhibitory signaling [378]. It was observed that a significant decrease in MMP-9 levels occurred after AHSCT in patients with MS [379]. In one study, normalization of apoptosis-related molecules resulted in therapeutic effects of AHSCT in MS patients [380]. Thus these studies highlight the emerging potential of HSCs in clinical MS.

### 2.9. Targeting iPSCs in MS

The iPSCs are a new area of research and hold a promising target in the treatment of many neurodegenerative disorders including MS. In a non-human primate (marmoset) model for progressive MS, hiPSCs derived OPCs after intracortical implantation; they specifically migrated toward the MS-like lesions in the corpus callosum where they myelinated the axons [381]. In a study, renal proximal tubule epithelial cells were isolated from MS patients urine and transfected with pluripotency-inducing episomal factors to give rise to iPSCs and eventually patient specific NPCs [382]. Transplantation of iPSC-derived NSCs significantly reduced T-cell infiltration and ameliorated white matter damage in the treated EAE mice indicating the effectiveness of iPSC-NSCs in treating MS [383]. The iPSCs have been used to generate OPCs that mature to form functional myelinating oligodendrocytes [384]. The iPSCs lines derived from MS patients (using defined factors Oct4, Sox2, Klf4, and c-Myc) successfully differentiated into mature astrocytes, oligodendrocytes, and neurons, thus representing a novel approach in the study of MS [385]. Human MAIT cells reprogrammed into iPSCs through Sendai virus harboring standard reprogramming factors efficiently redifferentiate into MAIT-like lymphocytes expressing the T-cell receptor V*α*7.2, CD161, and IL-18 receptor chain-*α* which then migrate to the bone marrow in MS [386]. Characterization of differentiation of iPSCs to oligodendrocytes involves several suppressive histone tags, deacetylation of lysine residues on histone H3, and trimethylation of residues K9 and K27, thus being relevant in MS [387]. The intrathecally transplanted iPSC derived NPCs after disease onset-ameliorated clinical and pathological features of EAE by exerting a neuroprotective effect through the secretion of LIF that promotes survival, differentiation, and remyelination capacity of both endogenous oligodendrocyte precursors and mature oligodendrocytes, thereby possessing therapeutic potential against MS [388]. OPCs derived from hiPSCs by forced expression of Sox10 and Olig2 differentiate into mature oligodendrocytes and thus have implications in MS [389].

### 2.10. Targeting ESCs in MS

ESCs prove to be a promising option for the generation and replacement of mature oligodendrocytes. Transplantation of ESCs expressing MOG antigen enhanced T-cell regeneration, long-term expression of MOG in the thymus, prevention of EAE development, and remission of established EAE [390]. Analysis of the changes in proteome pattern of EAE mouse model after ESCs derived NPCs transplantation demonstrated that the expression levels of various altered proteins in EAE samples returned to normal levels after transplantation, which implied a possible correlation between changes at the proteome level and decreased clinical signs of EAE in transplanted mice [391]. In EAE mice, transplanted hESCs derived OPCs were shown to generate TREM2-positive CD45 cells, amplified TIMP-1 expression, restricted inflammatory cells to the subarachnoid space, and augmented the number of Foxp3-positive regulatory T-cells, potentially providing new avenues for stem cell-based treatment of MS [392]. Intraspinal transplantation of embryoid body intermediate stage neural progenitor cells (EB-NPCs) into JHMV mice resulted in decreased accumulation of CD4^+^ T-cells in the CNS, thereby causing reduced demyelination. However, reduced neuroinflammation and remyelination were linked with a transient enhancement in CD4+FOXP3^+^ regulatory T-cells (Tregs) [393]. In hESCs derived OPCs, the addition of CNTF upregulated the Olig2 mRNA levels and this OPC population is shown to express Olig1/2, Sox10, PDGFR, NKX2-2, NKX6-2, oligodendrocyte-myelin glycoprotein, MBP, and PLP, ultimately maturing into oligodendrocytes [394]. The predicted targets of miRNAs expressed in oligodendrocytes from hESCs (involved in cellular differentiation and maintenance) include C11Orf9, CLDN11, MYTL1, MBOP, MPZL2, and DDR1, having roles in oligodendrocyte development and myelination [395]. Patients with MS treated with hESCs therapy showed an improvement in factors such as sleeping disorders, paralysis, muscle weakness, memory, language, irritability, eye pain, depression and coordination, communication, and appetite associated with MS [396]. These studies justify the role of ESCs in MS stem cell therapy.

### 2.11. Polyphenols Complementing Stem Cell Therapy and Reducing Neurodegeneration in MS

Many studies have highlighted the positive role of polyphenols in MS. Quercetin has been shown to ameliorate EAE by blocking IL-12-induced tyrosine phosphorylation of TYK2, JAK2, STAT3, and STAT4, leading to a decrease in IL-12-induced T-cell proliferation and Th1 differentiation [397]. Curcumin decreases T-cell production of IFN-*γ* [398], prevents the differentiation of Th1 cells [399], and decreases the amount of Th17- differentiated cells [400]. Resveratrol has been shown to induce neuroprotective effects through sirtuin 1 activation [401]. Lipopolysaccharide-activated primary rat astrocytes treated with flavonoids quercetin and catechins and the non-flavonoid resveratrol exert inhibitory effect on MMPs and represent a powerful tool for the downregulation of MMPs in the course of MS [402]. Nano-curcumin attenuated symptoms in EAE MS model, through downregulation of IL-17, NF-*κ*b, and TNF-*α* receptor expression and upregulation of anti-inflammatory genes and Nrf2, and increased MBP, Olig2, and PDGFR-*α* expression [403]. Polyphenolic white grape juice extract has been shown to neutralize the alteration of TNF-*α*, iNOS, Nitrotyrosine, PARP, Foxp3, Bcl-2, Caspase 3, and DNA fragmentation, thus acting as a novel tool in MS therapy [404]. The flavonoid-enriched fraction AF4 in mouse EAE MS model demonstrated disease recovery from EAE and reduced neuropathology in the cerebellum and spinal cord [405]. In a study on EAE mice, curcumin treatment significantly decreased elevated levels of IFN-*γ* and IL-17, IL-12, and IL-23, and this decrease was associated with upregulation of IL-10, PPAR-*γ*, and CD4^+^, CD25^+^, and Foxp3^+^ Treg cells, thus highlighting a differential regulation by curcumin [406]. The inhibition of MS by curcumin is mainly by the regulation of inflammatory cytokines such as IL-1*β*, IL-6, IL-12, TNF-*α*, IFN-*γ* and associated JAK-STAT, AP-1, and NF-kappaB signaling pathways [407]. Curcumin dramatically decreased IL-17, TGF-*β*, IL-6, IL-21, and STAT3 expression and also inhibited STAT-3 phosphorylation, thus suggesting neuroprotective role in EAE MS model [400]. The combined treatment of bone marrow-derived MSCs and resveratrol in EAE MS model was shown to suppress proinflammatory cytokines IFN-*γ* and TNF-*α* and increase anti-inflammatory cytokines IL-4 and IL-10, thus alleviating EAE symptoms [408]. Curcumin treatment of animals with EAE resulted in decrease in IL-12-induced STAT4 phosphorylation, IFN-*γ* production, and IL-12 Rbeta1 and beta2 expression and an increase in IFN-beta-induced STAT4 phosphorylation, IL-10 production, and IFN receptor (IFNAR) subunits 1 and 2 expression [409]. Resveratrol-mediated protection against EAE was found to be associated with rise in IL-17^+^/IL-10^+^ T-cells and suppression of macrophage IL-6 and IL-12/23 p40 expression [410]. Resveratrol significantly downregulated TNF-*α*, IFN-*γ*, IL-2, 9, 12, 17, MIP-1*α*, MCP-1, and RANTES in EAE MS model and, thereby, proved to be a useful tool against MS [411]. Similarly, curcumin treatment caused inhibition of JAK-STAT pathway which resulted in a decrease in IL-12-induced T-cell proliferation and Th1 differentiation; thus curcumin may serve as a potential polyphenol against MS [399]. In a cuprizone model of MS, resveratrol enhanced motor coordination and balance, reversed cuprizone-induced demyelination, improved mitochondrial function, alleviated oxidative stress, and inhibited NF-*κ*B signaling, while increasing Olig1 expression that is positively correlated to active remyelination [412]. Extra virgin olive oil and its polyphenols have been shown to improve MS disease symptoms [413]. Thus these studies provide new avenues as to how polyphenols cause neuroprotection against MS pathology and how they can utilize stem cells against MS regulating the same or different factors involved.

## 3. Mechanism of Action of Polyphenols for Neuroprotection

Neurodegenerative diseases such as AD and multiple sclerosis are multifactorial disorders possessing no single particular cause or pathophysiologic mechanism. However, chronic neuroinflammation is a common mechanism that is shared by multiple neurodegenerative diseases including AD and MS and is a general cause of the initiation and progression of neurodegeneration. The increase in inflammatory signaling results in an upregulation of nuclear factor-kappa B (NF-*κ*B) transcription factor [414, 415]. Increase in an abnormal accumulation of A*β*_1-42_ stimulates microglial cells and augments NF-*κ*B induced activation of inflammatory cytokines and neurotoxic molecules such as reactive oxygen species (ROS) and induced nitric oxide synthase (iNOS) [416]. Similarly, several NF-*κ*B polymorphisms are associated with an increased vulnerability to MS [414]. NF-*κ*B signaling plays a vital role in the activation of peripheral inflammatory cells and the CNS dwelling glial cells that eventually mediate inflammatory demyelination [414].

Polyphenols share a common mechanism of action for modulation of neuroinflammation by targeting the NF-*κ*B signaling [417]. Plant polyphenols including flavonoids inhibit the activation of NF-*κ*B through binding on toll-like receptor (TLR), specifically TLR4, thus reducing the chronic neuroinflammation [417]. In this line, tea polyphenol epigallocatechin-3-gallate has been reported to modulate the expression of Toll interacting protein. Resveratrol is shown to exert neuroprotective effects through oligomerization of TLR4 and activating TLR4/NF-*κ*B/STAT pathway [417]. Curcumin reduces the expression of inflammatory cytokines and neuronal apoptosis* via* inhibiting TLR4-MAPK/NF-*κ*B and TLR4/MyD88/NF-*κ*B signaling pathway [417]. Thus, polyphenol's mediated targeting of TLR4- NF-*κ*B signaling could be a potential therapeutic strategy for inhibiting neuroinflammation in AD and MS.

Apart from this common mechanism of action, polyphenols act through various receptors and pathways directly or indirectly. Resveratrol causes inhibition of MAPK and activation of SIRT1 pathway, thereby leading to suppression of NF-*κ*B signaling [418, 419]. In EAE, resveratrol ameliorates disease* via* apoptosis of activated T-cells and suppressing proinflammatory responses [410]. Demethoxycurcumin inhibits NF-*κ*B activation and suppresses proinflammatory gene expression in stimulated microglial cells [420]. Treatment with curcumin ameliorates EAE by preventing proinflammatory cytokine responses in microglial cells [399, 400]. EGCG interferes with IKK*β* activation and suppresses NF-*κ*B mediated activation of *β*-secretase, thereby inhibiting A*β* fibrillization [421].

Polyphenols are also known to modulate neurotrophic signaling pathways essential for neuronal growth, proliferation, differentiation, and survival [422]. Quercetin, resveratrol, and other polyphenols promote augmentation of neurotrophic factors such as NGF and BDNF [422]. The ERK pathway is involved in various physiological activities of neurons, including proliferation and differentiation [423]. Resveratrol and curcuminoids contribute to the regulation of ERK1/2 activation [424, 425]. A*β*-induced neuronal toxicity attenuates in response to activation of the ERK1/2 pathway [422]. PI3K and its downstream effector Akt are implicated in neuronal survival [422]. Polyphenols such as curcumin and ferulic acid are involved in improvement of A*β*-induced cognitive impairment and provide antidepressant effects, respectively, via the modulation of PI3K/Akt signaling [64] [426]. Polyphenols possess antioxidant properties and exert their antioxidant effects through activation of Nrf2 pathway, increase in expression of antioxidant enzymes, and induction of HIF-1-*α* pathway [427, 428]. Thus, these are the major common pathways that are utilized by polyphenols to exert their neuroprotective effects against neurodegenerative disorders such as AD and MS.

## 4. Conclusion and Future Perspectives

AD and MS are the most widespread neurodegenerative disorders characterized by the accumulation of abnormal proteins which give rise to the formation of pathogenic lesions. The severity of these disorders gradually increases with age of the individual. There exists no permanent treatment for these disorders. Therapies include anti-symptomatic drugs, monoclonal antibodies, stem cell therapy, and disease management. Studies are being carried out thoroughly on the use of stem cells as therapeutics against neurodegenerative disorders, and they are presenting promising results validating the safety and tolerability of stem cells in medicine. The use of therapeutic compounds coupled with stem cells for their stimulation is also being analyzed in regenerative medicine. Synthetic drugs and natural compounds such as polyphenols are being used for their potential stimulatory effect on stem cells against neurodegenerative diseases. The key focus of this review is, thus, to highlight the important factors involved in the pathogenesis of AD and MS and stem cell therapies currently utilized in the cure of these disorders and to appreciate the role of polyphenolic compounds in stimulating stem cell proliferation and differentiation as therapeutics against neurodegeneration.

AD is a complex neurodegenerative disorder involving abnormal accumulation of pathogenic amyloid beta species leading to impaired neurogenesis and neuronal death. The presence of mutant APOE *ε*4, PSEN1, and PSEN2 genes increases the susceptibility of A*β* accumulation in individuals and presents a genetic predisposition to AD. Apart from the genetic factor, exposure to environmental toxicants further aggravates the disease pathogenesis. Harmful exposure to toxic metals like lead and mercury, pesticides like rotenone and paraquat, and xenoestrogens like bisphenols results in elevated aggregation of pathogenic A*β*. This aggregation further impairs the physiological and metabolic functions of the body. Increased levels of apoptotic caspases and proinflammatory factors, elevation of ROS and oxidative stress, and dysfunction of autophagy and mitochondrial biogenesis are all end results of the AD pathophysiology ultimately causing neuronal loss and neurodegeneration. The rescue of the loss of neurons via promoting neurogenesis is promising, and this is where stem cell therapy comes into play. The utilization of endogenous and exogenous NSCs, ESCs, MSCs, and iPSCs has depicted promising results against AD. These stem cells either are engrafted directly or further release factors which in turn stimulate the endogenous NSCs population, thereby bringing about neurogenesis. Stimulation of factors such as BDNF, NGF, VEGF, FGF2, Oct4, Sox2, Pax6, MAP2, and DCX; regulation of Wnt/*β*-catenin, sonic hedgehog, Notch, and autophagy pathways; and activation of A*β* digesting enzymes such as neprilysin are central to stem cell-induced therapy. Although stem cells cause neurogenesis and neuroprotection in AD, there is wide appreciation of the role of polyphenols in regulating stem cell proliferation and differentiation. Curcumin, resveratrol, green tea extracts, quercetin, and epigallocatechin gallate have been shown to cause neuroprotection in AD* via* regulation of the above-mentioned NSC markers and stem cell proliferation and differentiation pathways and downregulation of the apoptotic and oxidative stress, thus complementing the stem cell therapy in the treatment of AD.

MS is another neurodegenerative disease, which is also caused by the accumulation of pathogenic proteins. It is more of an autoimmune disorder which ultimately leads to neuronal cell death. Genetic factors like presence of an abnormal HLA-DRB1*∗*15 gene, elevated apoptosis, and oxidative stress; increase in mitochondrial and ion-channel dysfunction; and exposure to environmental toxicants all lead to the pathogenesis of MS. Stem cell therapies complemented with polyphenol treatment have shown promising results in MS pathology although much work remains to be done. Quercetin, resveratrol, and curcumin have depicted positive results with MS by targeting the proinflammatory factors such as TNF-*α*, IFN-*γ*, and IL-2 and downregulating their expression. However, a great deal of research still needs to be carried out in studying the involvement of the role of polyphenols in regulating stem cells against MS.

The future perspectives involve further studies on the insights into mechanisms underlying the action of polyphenols in regulating stem cells against neurodegenerative disorders. A lot of polyphenols are still left unexplored, and very little research has been carried out to study their role in neurodegenerative disorders. Also, the role of polyphenols against other diseases such as disorders of the metabolism can be tested in case of neurodegeneration. Targeting of new molecular pathways and study of new physiological mechanisms need to be done pertaining to polyphenols and stem cells and how this can be useful in neurodegeneration. The notion of ineffectiveness of polyphenols due to natural origin needs to be abolished as these compounds hold potential promise in the area of medicine.

## Figures and Tables

**Table 1:** Genetic factors involved in AD.

**S. No.**	**Genetic Factors/Mutations**	**Effect in AD**	**References**
1.	R47H (Variant of TREM2)	It compromises microglial mediated clearance of aggregation-prone proteins in AD	[113]

2.	KIAA1462	It increases the risk of coronary artery disease, resulting in compromised blood flow to the brain, and increases oxidative stress and inflammation	[114]

3.	APOE *ε*4	ApoE regulates lipid homeostasis. *ε*4 allele increases the risk of AD by initiating and accelerating A*β* accumulation, aggregation, and deposition in the brain	[12]

4.	P21	P21 (cyclin-dependent kinase inhibitor) levels significantly decreased in peripheral blood lymphocytes of AD patients	[115]

5.	SORL1	SORL1 gene encodes a protein LR11 responsible for cargo transport, chaperone-like activity, signaling, and intracellular sorting. Down-regulation or dysfunction of LR11 has been shown to lead to amyloidogenesis.	[116]

6.	PSEN1	Presenilin-1 functions as the catalytic subunit of *γ*-secretase that cleaves APP. PSEN1 mutations initiate disease pathogenesis by increasing production of A*β*42.	[117]

7.	PSEN2	Presenilin-2 is the main component of the *γ*-secretase complex along with PSEN1. PSEN2 mutation alters the *γ*-secretase activity and increases A*β* 42/40 ratio.	[118, 119]

8.	CLU	The clusterin gene acts as an extracellular chaperone involved in lipid transport, complement regulation, apoptosis, endocrine secretion, and membrane protection. Elevated levels of CLU have been reported in the frontal cortex and hippocampus in AD.	[120–122]

9.	ABCA1	ABCA1 mediates cholesterol homeostasis, generation of phospholipids and immune system. The accumulation of A*β* is shown in the brain of ABCA7-deficient mice.	[123, 124]

10.	CR1	Complement Receptor-1 functions as a complement regulatory protein and helps in regulating the immune system. CR1 alteration is associated with increased cerebrospinal fluid (CSF) A*β*42 and vascular amyloid deposition.	[125, 126]

11.	CD33	CD33 mediates cell-cell interaction, inhibits immune cell functions and regulates cell growth and survival *via* promotion of apoptosis. CD33 contributes to the pathogenesis of AD by impairing microglia-mediated clearance of A*β*.	[127, 128]

12.	MS4A	MS4A regulates immunity and calcium influx. MS4A mediates AD pathogenesis *via* A*β* generation, tau phosphorylation, and apoptosis by altering calcium homeostasis.	[129, 130]

13.	BIN1	Bridging Integrator-1 regulates the immune response, synaptic vesicle endocytosis, apoptosis, intracellular APP trafficking and clathrin-mediated endocytosis. BIN1 knockdown suppresses tau-mediated neurotoxicity.	[131–133]

14.	CD2AP	CD2AP mediates regulation of the cytoskeletal structure, cell adhesion, receptor-mediated endocytosis, cytokinesis, apoptosis and intracellular trafficking. Suppression of CD2AP expression altered A*β* levels and decreased A*β*42/A*β*40 ratio.	[134, 135]

15.	PICALM	PICALM regulates intracellular trafficking and clathrin-mediated endocytosis. Knockdown of PICALM regulated APP internalization and decreased A*β* production, thus acting as a major regulator of brain A*β* production, APP internalization, and amyloid plaque load.	[136, 137]

16.	EPHA1	EPHA1 mediates synapse formation, chronic inflammation, and immune function. Association of EPHA1 variants with AD progression is well known.	[138, 139]

17.	INPP5D	INPP5D is a chief mediator of cytokine signaling and relates to increased late-onset AD.	[140]

18.	PTK2B	PTK2B is involved in activation of MAP kinase signaling pathway and calcium-induced regulation of ion channels. Fyn kinase inhibition of PTK2B improved the learning and memory impairment in AD mice.	[141, 142]

19.	SLC24A4/RIN3	SLC24A4/RIN3 is involved in neural development, lipid metabolism, and endocytic pathway. Association of SLC24A4 with methylation and brain DNA methylation is responsible for AD pathology.	[143–147]

20.	ADAM10	In Tg2576 AD mice, ADAM10 mutations were shown to increase plaque load and A*β* levels and decrease hippocampal neurogenesis.	[148]

21.	PLD3	Phospholipase D3 is highly expressed in the brain and is involved in epigenetic modifications, signal transduction, cell differentiation and neurotransmission. PLD3 is associated with APP processing and its over expression has been seen in AD patients. Accumulation of PLD3 on neuritic plaques in AD brains suggested its involvement in AD pathology.	[149–151]

22.	UNC5C	Neurons with UNC5C disease variant overexpression are more prone to A*β*-mediated cell death.	[152]

**Table 2:** Environmental factors involved in AD.

**S. No.**	**Environmental Factors**	**Effect in AD**	**References**
		**Toxic Metals**	

1.	Aluminium	It mediates AD neuropathy through oxidative stress, inflammation, neurofibrillary degeneration and cross-linking of A*β* oligomerization.	[153, 154]

2.	Copper	It induces A*β* plaques, oxidative stress, and neuroinflammation. Decreased Cu levels relate to reduced expression of cytochrome c, ceruloplasmin and superoxide dismutase in AD brain.	[155–158]

3.	Iron	Increased iron levels are associated with decreased tissue integrity in the hippocampus region of AD patients. Iron is involved in the formation of aggregates of A*β*-42 leading to the formation of amyloid fibrils.	[159, 160]

4.	Lead	Lead exposure increases APP expression, A*β* production, and hippocampal gliosis.	[161, 162]

5.	Cadmium	High cadmium levels have been shown in the hippocampus and cerebral cortex in AD patients. Cadmium leads to self-aggregation of the tau peptide R3 through neural and astrocyte cell toxicity, thereby causing AD.	[163, 164]

6.	Mercury	Mercury causes increased A*β* levels, tau protein hyperphosphorylation, reduced binding of GTP to *β*-tubulin, microtubule degeneration and compromised membrane structural integrity of neurites and neuron growth cones.	[165, 166]

7.	Arsenic	Arsenic exposure leads to tau hyperphosphorylation, increase in amyloid beta levels, increased generation of free radicals and increase in the inflammatory process.	[167]

8.	Selenium	High selenium concentration is associated with reduced cholinergic signaling, increased oxidative stress and degeneration of cholinergic neurons.	[168]

		**Insecticides and Pesticides**	

1.	Organochlorines Hexachlorocyclohexane (HCH) and Aldrin	HCH and Aldrin are two extremely persistent pesticides which undergo bioaccumulation. Increased blood levels of *β*-HCH have been associated with significant increase in AD risk, oxidative stress, and neurotoxicity.	[169]

2.	Organophosphates	Parathion causes morphological changes, affects non-cholinesterase targets like motor proteins, axonal transport, and the neuronal cytoskeleton.	[170]

3.	Carbamates	Carbofuran induces toxicity in neuronal nicotinic acetylcholinesterase receptors and reduces hippocampal neurogenesis, thereby leading to memory deficits.	[171–173]

4.	Bipyridyl	Paraquat induces mitochondrial dysfunction in cerebral cortex, thereby promoting impairment of cognitive function with elevated levels of A*β*.	[174]

5.	Rotenone	Rotenone induces mitochondrial dysfunction leading to AD.	[175]

6.	Fipronil	It is a phenylpyrazole insecticide which is known to show functional remodeling of GABAergic neurotransmission in AD patients, thereby causing AD.	[176]

7.	Pyrethroid	It induces imbalanced tau phosphorylation, cognitive abnormalities and AD-like pathology in rats.	[177]

		**Industrial and Commercial Pollutants**	

1.	Polybrominated Diphenyl Ethers (PBDEs)	PBDEs are neurotoxic and amyloidogenic, causing amyloid-*β* peptide release, apoptosis, Ca^2+^ ATPase inhibition, reduced hippocampal cholinergic receptors and impaired learning and memory.	[178, 179]

2.	Octylphenol	Octylphenol increases expression of amyloid-like precursor protein-2 and APP leading to AD in turtles.	[180]

3.	Tetrachlorodibenzo-p-dioxin (TCDD)	TCDD increases calcium levels and tau phosphorylation *via* up-regulation of p-GSK3*β*, thereby causing AD in patients.	[181]

4.	Bisphenol A	BPA causes an alteration in behavior, inhibition of spine synapse formation in the prefrontal cortex and hippocampus, thereby having implications in AD. BPA exposure alters autophagy, causes mitochondrial dysfunction and myelin dysfunction and reduces neurogenesis leading to cognitive decline in rats.	[83, 182–188]

		**Anti-Microbials**	

1.	Triclocarban	Triclocarban impairs neurogenesis, neurobehavioral development and disrupts hormone signaling pathways.	[189]

2.	Triclosan	Triclosan has been shown to cause neurotoxicity, altered neurodevelopment and neuroplasticity through disruption of the brain Ca^2+^ homeostasis by altering ryanodine (Ry) receptor type 1.	[190]

3.	Parabens	Paraben exposure has been shown to cause reduced neurotransmitter activity, behavioral changes, neurodevelopmental disturbances and neurotoxicity in animal studies.	[191]

**Table 3:** Physiological factors involved in AD.

**S. No**	**Factors**	**Effect in AD**	**References**
1.	Apoptosis	(i) Studies have shown extracellular and intracellular A*β* mediated activation of caspases either through the extrinsic or the intrinsic pathway.	[192, 193]
		(ii) A*β* binding to alcohol dehydrogenase can activate mitochondrial stress mediated apoptosis.	[194]
		(iii) Caspases and Calpains are proteases responsible for Tau proteolysis, and their activation has been found to play a role in apoptosis.	[195, 196]
		(iv) The lysosomal protease Cathepsin D expressed in the brain regulates apoptosis, thus contributing to AD.	[197]
		(v) P_2_X_7_, a purinoreceptor involved in AD pathogenesis promotes cell death by apoptosis.	[198]
		(vi) Altered expression of caspase-3, Bax, p53, Bcl-2 and Par-4 apoptotic proteins occurs in AD.	[199]
		(vii) TRAIL binding to death receptor 5 (DR5) has been shown to initiate Caspase-8 mediated apoptosis in AD neurons.	[200]
		(viii) A*β*-induced synthesis of disialoganglioside GD3 is involved in apoptosis induction in cortical neurons.	[201]
		(ix) In neurons, over-expression of RCAN_1-1_ initiates A*β* mediated apoptosis through activation of caspase-9 and caspase-3. An additional copy of RCAN_1_ on chromosome 21 promotes AD pathogenesis.	[199]
		(x) Regulation of AICD mediated neuronal apoptosis occurs via GSK3*β* and p53.	[202, 203]
		(xi) PSEN1 mutants exhibit increased A*β* generation, altered calcium homeostasis and an augmented sensitivity to ER stress-induced apoptosis.	[204]
		(xii) Increased caspase-4 in AD brains relates to ER stress-induced apoptosis.	[205]

2.	Autophagy	(i) Increased levels of lysosomal protease occur in AD patients.	[206]
		(ii) In Drosophila melanogaster, age-related down-regulation of expression of atg1, atg8a and atg18 was associated with late onset of AD.	[207]
		(iii) In ATG7 knockdown mice, significant reduction in A*β* secretion was followed by intracellular A*β* accumulation.	[208]
		(iv) The RAGE-calcium-CaMKK*β*-AMPK pathway was found to regulate the A*β*-induced formation of autophagic vacuoles.	[209]
		(v) Tau hyperphosphorylation is implicated in autophagy dysfunction.	[210, 211]
		(vi) Tau degradation has been found to be regulated by Nrf2-mediated activation of NDP52 autophagy receptor.	[212]
		(vii) Beclin-1 deficiency resulted in an elevation of APP, A*β* and the C-terminal fragment (CTF), while its over-expression caused a reduction in A*β* accumulation.	[213–215]
		(viii) Rapamycin has been shown to reduce A*β* and Tau pathology and improve cognition.	[216, 217]

3.	Oxidative Stress	(i) A decrease in reduced glutathione (GSH) causes excess ROS production leading to oxidative stress, thus favoring AD pathogenesis.	[218]
		(ii) A*β* induces lipid peroxidation and its products.	[219]
		(iii) Breakdown products of oxidative stress, such as malondialdehyde, acrolein, F2-isoprostanes and 4-hydroxy-2,3-nonenal (HNE), are observed in AD brains.	[220–223]
		(iv) Localization of increased levels of 8OHdG and 8OHD (associated with DNA and RNA oxidation) in A*β* plaques and NFTs is shown.	[224–227]
		(v) In AD, accumulation of extracellular advanced glycation end products (AGEs) occurs due to increased oxidation of glycated proteins.	[228]

4.	Mitochondrial Dysfunction	(i) A significant increase in mtDNA and cytochrome oxidase (COX) in AD neurons is reported.	[229–231]
		(ii) Association of mitochondrial pathology in AD with loss of dendritic branches, dystrophic dendrites and abnormal alteration of the dendritic spines is evident.	[232, 233]
		(iii) C57B6/SJL Tg AD mice showed increased levels of mtDNA deletion, amyloid deposition, mitochondrial structural abnormalities and oxidative stress markers.	[234, 235]
		(iv) In AD, accumulation of A*β*PP across mitochondrial import channels restricts entry of COX subunits IV and Vb leading to a decrease in COX activity and elevated H_2_O_2_ levels.	[236]
		(v) In the triple Tg AD mouse model, increased levels of lipid peroxidation, GPx, and SOD, but decreased levels of vitamin E and GSH, were observed.	[237]
		(vi) A *γ*-secretase complex, composed by PEN2, APH-1, and NCT, was identified in the rat brain mitochondria and was shown to cleave A*β*PP into A*β* and A*β*PP intracellular domain.	[238]
		(vii) In AD, a decrease in dynamin-like protein 1 (DLP1) and OPA1 and increase in Fis1 levels, thereby leading to mitochondrial abnormalities, have been reported.	[239, 240]
		(viii) Studies show that A*β* peptides in the presence of Ca^2+^ aggravate opening of mitochondrial PTP.	[241, 242]
		(ix) In PC12 cells exposed to A*β*_40_ and A*β*_25–35_, inhibition of complexes I, III, and IV of the mitochondrial respiratory chain was observed, thereby leading to mitochondrial dysfunction.	[243]

5.	Inflammation	(i) Associations between AD pathogenesis and mutations in TREM2, CD33 have been established.	[127, 244, 245]
		(ii) A*β* binding to CD36 or TLR4 leads to the production of inflammatory cytokines.	[246, 247]
		(iii) In AD animal models, elevated levels of pro-inflammatory markers IL-1, IL-6, GM-CSF, IL-23, IL-12, and TNF were detected.	[248–251]
		(iv) Microglia from Tg AD mice has shown reduced A*β*-binding scavenger receptor and A*β*-degrading enzyme levels.	[252]
		(v) Anti-inflammatory factors in neurons such as CD200, CD59, and fractalkine have been shown to be down-regulated in AD brains.	[253–256]
		(vi) In the human AD brain, endothelial cells have been shown to produce IL-1*β*, CCL2, and IL-6 immune molecules.	[257]

**Table 4:** Factors involved in the pathogenesis of MS.

**S. No.**	**Factors **	**Involvement in MS**	**References**
1.	Genetics	(i) HLA-DRB1*∗*15 is the most common genetic risk factor for MS.	[258, 259]
		(ii) GWAS have predicted GRIN2A, encoding NR2A subunit of NMDA-type glutamate receptors a possible candidate in MS.	[260, 261]

2.	Oxidative Stress	(i) ROS and RNS such as nitric oxide (NO) produced by macrophages and microglia in MS lesions lead to inflammation.	[262–264]
		(ii) In MS lesions, microglia has shown up-regulation of NOX1, NOX2, and NOX organizer 1 enzymes responsible for ROS generation.	[265]
		(iii) Iron accumulation in MS patients further amplified ROS and RNS-mediated injury through the generation of toxic reactants.	[266, 267]
		(iv) NRF2 mediated HMOX1 anti-oxidant enzyme levels are known to increase in MS lesions.	[268, 269]
		(v) Dimethyl fumarate (DMF) induces Nrf2 expression in neurons, thus contributing to attenuation of MS in patients.	[270]

3.	Mitochondrial Dysfunction	(i) Altered mitochondrial transport, mediated by translocation of HDAC1 from the nucleus to the axoplasm, thereby hindering kinesin motor protein functions, is evident in experimental autoimmune encephalomyelitis (EAE) lesions model of MS.	[263, 271]
		(ii) Resveratrol-mediated attenuation of neuronal damage in optic neuritis in EAE is brought about by activating SIRT1, thus promoting mitochondrial function.	[272, 273]
		(iii) Mitochondrial permeability transition (MPT), which is dependent on cyclophilin D (CyPD), results in equilibration of ionic gradients, loss of mitochondrial transmembrane potential and termination of oxidative phosphorylation followed by necrosis.	[274]
		(iv) Due to impaired mitochondrial function in MS hypoxia develops which causes nuclear translocation of HIF1*α* and its subsequent activation.	[275–277]

4.	Ion Channel Dysfunction	(i) Increased Na^+^ concentration associates with MS pathology.	[278]
		(ii) Presence of voltage-gated Na^+^ channel (Na_v_)1.2, Na_v_1.6 and Na^+^/Ca2^+^ exchanger (NCX) subunits is evident in MS lesions.	[279]
		(iii) Altered expression or activation of voltage-gated K^+^ (K_v_) channels, which is evident in MS, is blocked by 4-aminopyridine (non-specific blocker of K_v_ channels) in MS patients.	[280]
		(iv) Altered glutamate levels result in excessive neuronal signaling, thereby leading to Ca^2+^ mediated excitotoxicity as evident in MS.	[281]
		(v) The glutamate-mediated axonal injury increases by a reduction in mitochondrial complex IV activity, thereby contributing to EAE pathology.	[282]
		(vi) Abnormal NMDA receptor function contributes to dysfunctional mitochondrial activity. Thus, inhibition of NMDA and AMPA receptors leads to improvement in EAE.	[283–285]
		(vii) Inhibition of calpains (Ca^2+^- dependent proteases involved in degradation of axonal components) improved EAE pathology.	[286]

5.	Apoptosis	(i) Mice overexpressing the antiapoptotic Bcl-2 protein showed attenuated EAE severity and reduced axonal loss.	[287]
		(ii) Cytokine TRAIL can induce caspase-dependent apoptosis in neurons by binding to death receptors DR4 and DR5.	[288]
		(iii) Wallerian degeneration is involved in axon loss in MS patients. Expression of Wallerian degeneration slow (Wld^s^) protein inhibits this process through decreased microglial and macrophage activation levels and increased expression of CD200 glycoprotein, which inactivates monocytes by binding to the CD200 receptor, thereby providing protection.	[289, 290]

6.	Environment	(i) Genes that encode proteins involved in the actions of vitamin D associate with the risk of developing MS. Polymorphisms in 1*α*-hydroxylase encoding gene CYP27B1 are coupled with an increased risk of developing MS.	[291]
		(ii) Infection with Epstein Barr virus (EBV) in association with infectious mononucleosis (IM) is linked with increased MS risk. Increased MS risk explicitly associates with higher IgG antibody titers to Epstein-Barr nuclear antigens (EBNA).	[292–294]
		(iii) An increased MS risk associated with the interaction between smoking and high anti-EBNA titers is observed.	[295]
